# Health-related quality of life and mental health of adolescents with cerebral palsy in rural Bangladesh

**DOI:** 10.1371/journal.pone.0217675

**Published:** 2019-06-11

**Authors:** Rosalie Power, Mohammad Muhit, Eamin Heanoy, Tasneem Karim, Nadia Badawi, Rahena Akhter, Gulam Khandaker

**Affiliations:** 1 Discipline of Child and Adolescent Health, Sydney Medical School, University of Sydney, Sydney, New South Wales, Australia; 2 Asian Institute of Disability and Development (AIDD), University of South Asia, Dhaka, Bangladesh; 3 CSF Global, Dhaka, Bangladesh; 4 Cerebral Palsy Alliance Research Institute, University of Sydney, Sydney, New South Wales, Australia; 5 Faculty of Dentistry, University of Sydney, Sydney, New South Wales, Australia; 6 Public Health Unit, Central Queensland Hospital and Health Service, Rockhampton, Queensland, Australia; IRCCS E. Medea, ITALY

## Abstract

**Aim:**

To assess the health-related quality of life (HRQoL) and mental health of adolescents with cerebral palsy (CP) in rural Bangladesh.

**Methods:**

Case-control study of adolescents with CP (10 to ≤18-years) and age and sex matched controls without disability. Primary caregivers were included for proxy report. HRQoL was measured with Bengali versions CP Quality of Life-Teens (CPQoL-Teens) and KIDSCREEN-27. Mental health was measured with Strengths and Difficulties Questionnaire (SDQ).

**Results:**

154 cases and 173 controls matched on age and sex participated (mean age 15.1 (1.6) and 14.9 (1.6) respectively; female *n* = 48, *n* = 55 respectively, *p*>0.05). CPQoL-Teens was administered to adolescents with CP only; mean outcomes ranged from 38.5 (27.4) to 71.5 (16.1) and ‘feelings about functioning’ was poorest domain for both self- and proxy-report groups. KIDSCREEN-27 was administered to adolescents with CP and controls; adolescents with CP mean outcomes ranged from 25.9 (12.2) to 48.7 (10.56) and were significantly poorer than controls, mean difference 4.3 (95% CI 0.7 to 7.8) to 16.7 (95% CI 14.5 to 18.5), *p*<0.05. ‘Peers and social support’ was poorest domain for all groups. In regards to mental health, adolescents with CP reported significantly poorer mean SDQ than peers without disability, mean difference 0.7 (95% CI 0.3 to 1.1) to 7.8 (95% CI 6.7 to 8.9), *p*<0.05; and were for self-report 7.8 (95% CI 2.6 to 23.0) and proxy-report 12.0 (95% CI 6.9 to 20.9) times more likely to report ‘probable’ range ‘total difficulties’ score. Individual item analysis of CPQoL-Teens and KIDSCREEN-27 identified unique areas of concern for adolescents with CP related to pain, friendships, physical activity and energy, what may happen later in life, and feelings about having CP. Financial resources were of concern for both cases and controls.

**Interpretation:**

Adolescents with CP in rural Bangladesh are at high risk of poor HRQoL and mental health problems. Effort to reduce the disparity between adolescents with CP and those without disability should consider wellbeing holistically and target dimensions including physical, psychological and social wellbeing. Specific interventions to alleviate modifiable aspects of HRQoL including pain, social isolation, and physical in-activity are recommended.

## Introduction

Cerebral palsy (CP) refers to a group of disorders affecting a person’s ability to move that is caused by damage to the developing brain either during pregnancy or shortly after birth [1]. CP is considered to be one of the major causes of childhood disability. Globally, there is an estimated 17 million people living with CP of which prevalence is thought to be five to ten times higher in low and middle-income countries (LMICs) like Bangladesh [2]. Bangladesh is the eight most populous country in the world and has a large adolescent population constituting nearly one-fifth of the country’s total population [3]. A recent population-based study in Bangladesh reported the prevalence of CP to be 3.4 per 1,000 children [4]; equating to more than 90,000 adolescents with CP. 68.2% of the children with CP in Bangladesh were unable to walk and more than half had cognitive or speech impairments. Moreover, rates of epilepsy, visual and hearing impairment were above international norms [4, 5]. CP, is a lifelong condition that in contexts where disability is viewed as non-normative and ‘able-bodies’ are favoured has potential to negatively affect health-related quality of life (HRQoL) and mental health.

HRQoL is a subjective multidimensional concept for measuring the interaction between health status and physical, psychological, and social aspects of wellbeing [6]. HRQoL is an important outcome measure in clinical interventions and treatment, health service evaluation, understanding burden of disease, allocation of health resources, and policy guidance [7]. Measure of HRQoL of adolescents with CP from LMICs is pertinent; multi-national research from high income countries (HICs) has shown similar HRQoL amongst adolescents with CP and their peers without disability, with exceptions of ‘social support and peer’ dimensions [8] whereas research from LMICs has shown poor HRQoL and large disparities to peers [9]. A recent systematic review on HRQoL among children with CP in LMICs showed that dimensions of physical wellbeing were often poorest overall and correlated to child’s level of motor functioning [9]. Moreover, the review identified numerous gaps in HRQoL research from LMICs. The review recommended future HRQoL research in LMICs to incorporate multi-respondent assessment, both general population and CP-specific tools for measuring HRQoL and conduct of analysis by age group to delineate the HRQoL of adolescents as a unique cohort.

The mental health of adolescents with CP is also critical. The interaction of numerous physiological and social factors can affect mental health during adolescence including changes related to puberty, family and social relationships, and current and future opportunities and expectations (i.e. related to independence and employment) [10]. Moreover, adolescents with CP are at greater risk of emotional and behavioural problems than those without disability and typically report poor overall psychological wellbeing; although the mental health status of adolescents with CP in LMICs, including Bangladesh, is largely unknown [11].

Bangladesh, a typical LMIC, is at a critical stage of disability infrastructure and health service development [3, 12]. Throughout the past decade ideological changes have resulted in increased recognition of the rights of persons with disability and subsequent policy focus, advocated for by people with disability and their allies, have led to positive change [13]. However, people with disability continue to be significantly more likely to live in poverty, lack access to education and health services, be socially isolated, and experience high vulnerability to poor reproductive and sexual health including sexual abuse [12]. Adolescents with CP are particularly vulnerable as they negotiate these challenges alongside transition to adulthood; the impact of which will be more encompassing for those in rural areas whereby infrastructure and service development is often the poorest [14].

Comprehensive measurement of the HRQoL and mental health of adolescents with CP is necessary to guide resource allocation and intervention planning and ensure service development aligns with principles of the United Nations Convention on the Rights of Persons with Disabilities (CRPD) and Sustainable Development Goals (SDG), of which Bangladesh is an adoptee. In this study we assessed the HRQoL and mental health of adolescents with CP in rural Bangladesh, compared wellbeing to age and sex matched peers without disability, and made recommendations for priority areas for resource allocation.

## Methods

### Study design and participants

This is a matched case-control study. Cases (adolescents with CP aged 10 to ≤18 years) were identified using the Bangladesh Cerebral Palsy Register (BCPR) [4, 14]. BCPR is the first population-based register of children and adolescents with CP in an LMIC. The register holds data on socio-demographic, clinical (including severity, aetiology, associated impairments and risk factors), nutrition, education and rehabilitation status of children and adolescents with CP in Bangladesh. Participants were considered as adolescents from 10 years of age; this is a normative classification of adolescence in Bangladesh [15].

BCPR uses Key Informant Methodology described in Khandaker et al. [14] to identify children and adolescents with CP in Bangladesh. The register has been operating since January 2015 and covers a defined, representative, geographical region of the Shahjadpur sub-district of Sirajganj district in the northern part of Bangladesh. For the present study we attempted to contact all adolescents aged 10 to ≤18 years registered with the BCPR. We also requested participation from their primary caregiver classified as a parent, grandparent, other relative or close adult friend who provided the majority of their care and support. The control group of adolescents without disability were identified using convenience sampling. Adolescents living in dwellings neighbouring participants with CP were screened for matching age, sex and absence of impairments and invited to participate.

Informed verbal and written consent was obtained for all individual participants included in the study. Verbal consent was obtained for all minors (<16 y) and then written consent was obtained from their parent or legal guardian. In cases where adolescents were unable to provide verbal consent (i.e. due to severe communication impairment or perceived lack of capacity) then consent was only obtained from the parent or legal guardian and data was only collected as proxy data. No data was collected in instances that adolescents indicated objection to participation, even in instances of parental consent. In cases of illiteracy, written consent was obtained by thumbprint. This study adhered to STROBE guidelines and all methods described adhered to the ethical approvals provided by the Bangladesh Medical Research Council (BMRC/NREC/2013-2016/1165) and University of Sydney Human Research Ethics Committee (2016/646).

### Measures

Adolescent self- and proxy-reported (via primary caregivers) data was collected from all participants. Adolescents were excluded from self-reporting if they appeared unable to understand questions or communicate answers. In these instances only proxy-reported data was captured. The questionnaires were interviewer-administered.

#### Cerebral Palsy Quality of Life Questionnaire for Teens

We administered the Cerebral Palsy Quality of Life Questionnaire for Teenagers (CPQoL-Teens) to adolescents with CP and their proxies (i.e. cases only). CPQoL-Teens measures quality of life in 13 to 18 year olds with CP, has self- and proxy-report versions, and uses a nine point Likert scale to assess ‘general wellbeing and participation’, ‘communication and physical health’, ‘school wellbeing’, ‘social wellbeing’, ‘feelings about functioning’, ‘access to services’ and ‘family health’. CPQoL-Teens was originally developed and applied in Australia [16, 17]. We cross-culturally translated (i.e. to Bengali) and validated the questionnaire for use in Bangladesh. The questionnaire reported good psychometric properties [18].

#### KIDSCREEN-27

We administered KIDSCREEN-27 to all adolescents and proxies (i.e. cases and controls). KIDSCREEN-27 measures HRQoL in 8 to 18-year olds. The instrument has self- and proxy-report versions and uses Rasch scales to measure participant subjective perception of their health and wellbeing over the last week in relation to; ‘physical wellbeing’, ‘psychological wellbeing’, ‘autonomy and parents’, ‘peers and social support’, and ‘school environment’. KIDSCREEN was originally developed simultaneously in 13 European counties [19] and has been shown to function in a similar way in children with CP as in the general population [20]. We cross-culturally translated (i.e. to Bengali) and validated the questionnaire for use in Bangladesh; the questionnaire reported good psychometric properties described in Power et al [21].

#### Strengths and Difficulties Questionnaire

We administered the Strengths and Difficulties Questionnaire (SDQ) to all adolescents and proxies (i.e. cases and controls). SDQ is a brief behavioural screening questionnaire for identifying mental health and wellness of 4 to 17 year olds. SDQ assesses emotional symptoms, conduct problems, hyperactivity/ inattention, peer relationship problems, and pro-social behaviour. SDQ has previously been translated and validated for use in Bangladesh [22].

#### Data extracted from the Bangladesh Cerebral Palsy Register

We extracted demographic and impairment related information about participants from the BCPR such as age, sex, Gross Motor Functional Classification System (GMFCS) level, associated impairments (other than CP), service access and school attendance. Children classified at GMFCS level I-II are independently ambulant whereas children ranked Level III-V require wheeled mobility [23].

#### Statistical methods

Data was checked for accuracy and records where age was invalid (≤9 and ≥19 years at date of data collection) were excluded from analysis. CPQoL-Teens scores were converted to values between 0 to 100 and KIDSCREEN-27 scores were converted to T-values (standardised mean = 50, SD = 10). CPQoL-Teens and KIDSCREEN-27 mean dimension scores were calculated by averaging the items in each dimension. KIDSCREEN-27 ‘Total score’ was calculated as an average of each dimension; dimensions with missing scores were weighted. SDQ scores were summed into dysfunction scales (range = 0 to 10). Total difficulties score was computed by summing the emotional, conduct, hyperactivity and peer problems scales (range 0 to 40). Higher scores indicated better HRQoL, for CPQoL-Teens and KIDSCREEN-27, and better mental health for SDQ. Outcomes were assessed for normality using Shapiro-Wilk and visual inspection of residual plots. Differences between groups were assessed with independent samples *t*-tests, Mann-Whitney U, chi-square test of homogeneity and fishers exact test. Odds ratio was calculated for SDQ outcomes. All statistical analysis was conducted using SPSS version 24 (IBM Corporation, Chicago, Illinois, USA). A *p* value of <0.050 was considered significant.

## Results

### Participant characteristics

Among 192 adolescents with CP registered into the BCPR, 154 enrolled in this study (participation rate 80.2%). Reasons for non-participation included being unwilling to participate (*n* = 11); no longer living in the surveillance area (*n* = 7); not able to be retraced (*n* = 17); and having deceased (*n* = 3).

Mean age of the 154 adolescents with CP was 15.1 (1.6) (range 10-18y) of which 48 (31.2%) were female. Controls were 173 peers without disability (mean age 14.9 (1.6), range 10-18y) of which 55 (31.8%) were female. Adolescents with CP matched controls by age and sex (*p*>0.05); see Table 1. Adolescents with CP were less likely to attend school (*p*<0.001).

**Table 1:** Characteristics of adolescents with CP and their age and sex matched peers without disability (i.e. controls).

Sociodemographic characteristic	Adolescents with CP	Controls	
	*n* = 154	*n* = 173	*p-value*
**Age (years)**			
Mean (SD)	15.1 (1.6)	14.9 (1.6)	0.282
Median (IQR)	14.6 (2.6)	15.0 (2.3)	
**Sex** (n,%)			
Female	48 (31.2%)	55 (31.8%)	0.904
Male	106 (68.8%)	118 (68.2%)	
**School attendance** (n,%)			
No	115 (74.7%)	6 (3.5%)	**<0.0001**
Yes	39 (25.3%)	167 (96.5%)	

64 adolescents with CP provided both self- and proxy-reported data. 90 adolescents with CP were unable to self-report and provided only proxy-reported data (i.e. adolescent with CP total self-report *n* = 64, total proxy-report *n* = 154), see Table 2. All controls (i.e. 100%) provided self- and proxy-reported data. Proxy reporters were mothers (cases *n* = 118; controls *n* = 119), fathers (cases *n* = 21; control *n* = 7) and other primary caregivers (cases *n* = 15; control *n* = 47).

**Table 2:** Characteristics of adolescents with CP according to reporting method.

Sociodemographic characteristic	Adolescents with CP		*p-value*
	Self- and proxy-report *n* = 64	Proxy-report only (i.e. excluded from self-report) *n* = 90	
**Age (years)**			
Mean (SD)	15.3 (1.7)	15.0 (1.6)	0.387
Median (IQR)	14.9 (2.9)	14.5 (2.4)	
**Sex** (n,%)			
Female	15 (23.4%)	33 (36.7%)	0.112
Male	49 (76.6%)	57 (63.3%)	
**GMFCS** (*n*,%)			
Level I	14 (21.9%)	22 (24.4%)	**<0.001**
Level II	13 (20.3%)	10 (11.1%)	
Level III	23 (35.9%)	10 (11.1%)	
Level IV	10 (15.6%)	10 (11.1%)	
Level V	3 (4.7%)	38 (42.2%)	
Unknown	1 (1.6%)	0	
**Associated impairment** (n,%)			
Epilepsy	8 (12.5%)	28 (31.1%)	**0.007**
Cognitive impairment	18 (28.1%)	69 (76.7%)	**<0.001**
Visual impairment	3 (4.7%)	9 (10%)	0.361
Hearing impairment	4 (6.3%)	15 (16.7%)	0.080
Speech impairment	20 (31.3%)	83 (92.2%)	**<0.001**
**School attendance** (n,%)			
No	31 (48.4%)	84 (93.3%)	**<0.001**
Yes	33 (51.6%)	6 (6.7%)	

Adolescents with CP who self-reported were more likely than those with only proxy-report to have lower GMFCS level (*p*<0.001), less likely to have epilepsy (*p* = 0.007), cognitive impairment (*p*<0.001), or speech impairment (*p*<0.001); and were more likely to have gone to school (*p*<0.001).

### HRQoL and mental health outcomes

HRQoL outcomes according to instrument dimensions are shown in Tables 3, 4 and 5. All dimensions reported full scores, except ‘school’ for CPQoL-Teens and KIDSCREEN-27 (missing cases self-report 48.4%, proxy-report 74.7%; controls self-report 12.1%, proxy-report 11.0%). Missing scores corresponded approximately to rates of non-school attendance, explained by non-acceptance from school due to impairment (65.6%), parent refusal (3.2%), school too far away or lack of transport (3.9%), lack of money (0.6%), and adolescent already employed (0.6%). GMFCS level, cognitive and speech impairments moderately correlated to non-school attendance (*r* -0.372, -0.362, -0.479, respectively, *p*<0.010).

**Table 3:** CPQoL-Teens mean scores of adolescents with CP. Higher scores indicate better HRQoL (range 0–100).

*CPQoL-Teens dimensions*	Self-report *n* = 64	Proxy-report *n* = 154
	Mean (SD)	Mean (SD)
General wellbeing and participation	62.0 (17.4)	45.2 (21.3)
Communication and physical health	65.2 (15.0)	49.0 (17.9)
School wellbeing	71.5 (16.1)^b^	63.5 (18.3)^c^
Social wellbeing	74.3 (14.3)	59.9 (19.8)
Feelings about functioning	57.7 (25.5)	38.5 (27.4)
Access to services	-^a^	47.6 (25.2)
Family health	-^a^	51.4 (23.2)

^a^ Proxy-report only;

^b^ *n* = 33;

^c^ *n* = 39

**Table 4:** CPQoL-Teens individual item analysis by proportion of adolescents with CP rating ‘unhappy’ or ‘very unhappy’ (i.e. ≤ 3 where 1 = very unhappy and 9 = very happy).

CPQoL-Teens Item	Self-report	Proxy-report
**General wellbeing and participation**		
Your life in general?	35.9%	64.9%
Your life as a whole?	40.6%	67.5%
Your quality of life?	39.1%	64.9%
The way you get along with other teenagers outside of school (not school friends)?	15.6%	31.8%
Hanging out on your own?	62.5%	65.6%
Hanging out with friends?	9.4%	33.8%
The way you are accepted by other teenagers outside of school?	20.3%	35.7%
Doing things you want to do?	6.3%	24.7%
Having a go and trying new things?	15.6%	42.2%
Yourself?	32.8%	52.6%
Your positive attitude?	12.5%	37.7%
Your future?	42.2%	71.4%
Your opportunities in life?	40.6%	53.2%
Your ability to participate in leisure and recreational activities?	32.8%	48.7%
Your ability to participate in sporting activities?	34.4%	53.9%
Your ability to participate in social events outside of school?	32.8%	51.9%
Your ability to participate in the community?	29.7%	51.9%
The way you get around?	37.5%	49.4%
Succeeding in things you want to be good at?	10.9%	27.3%
Your ability to get around in your neighbourhood?	14.1%	27.9%
Your ability to get from place to place?	29.7%	39.0%
**Communication and physical health**		
The way you get along with adults?	15.6%	31.8%
The way you are accepted by adults?	7.8%	26.6%
The way you are accepted by people in general?	17.2%	35.7%
Your ability to keep up academically?	9.4%	10.4%
Your ability to communicate with people you know well?	9.4%	22.1%
Your ability to communicate with people you do not know well?	35.9%	50.0%
The way other people communicate with you?	14.1%	28.6%
The way you communicate with people using technology?	21.9%	39.0%
Your overall health?	29.7%	55.8%
Your physical health?	29.7%	58.4%
How you sleep?	15.6%	33.8%
Changes happening to your body to do with puberty?	21.9%	44.2%
Being able to do things by yourself without relying on others?	21.9%	48.7%
What may happen to you later in life?	51.6%	72.1%
What you have achieved in your life?	28.1%	56.5%
Your plans for the future?	43.8%	64.9%
**School wellbeing**		
The way you get along with other teenagers at school?	1.6%	5.2%
The way you are included by other students at school?	9.4%	5.2%
The way you get along with your teachers at school?	0.0%	3.2%
The way you are accepted by other students at school?	1.6%	3.2%
The way you are accepted by staff and teachers at school?	6.3%	1.3%
The way you are treated the same as everyone as at school?	9.4%	5.8%
Your ability to keep up physically?	20.3%	16.9%
Your ability to participate at school?	12.5%	9.1%
**Social wellbeing**		
How happy you are?	26.6%	55.2%
The way you get along with people generally?	12.5%	40.9%
The way you get along with your parents?	1.6%	24.7%
The support they get from your family?	3.1%	14.9%
The way they get along with your brothers and sisters?	4.7%	17.5%
Going out on trips with the family?	17.2%	25.3%
The way you are accepted by their family?	4.7%	9.1%
**Feelings about functioning**		
The way you use your arms and hands?	34.4%	50.0%
The way you use your legs?	56.3%	61.0%
Your ability to dress yourself?	25.0%	57.1%
Your ability to eat or drink independently?	17.2%	48.7%
Your ability to use the toilet by yourself?	35.9%	59.1%
**Access to services**		
How much pain does your teenager have? ^a^	95.1%	82.3%
Your teenagers access to treatment?	-	54.5%
Your teenagers access to physiotherapy?	-	59.1%
Your teenagers access to speech therapy?	-	64.9%
Your teenagers access to occupational therapy?	-	66.2%
Your teenagers access to specialised medical or surgical care?	-	65.6%
Ability to get advice from a paediatrician?	-	58.4%
Your teenagers access to community services and facilities?	-	44.8%
Your teenagers access to extra help with learning at school?	-	22.1%
**Family health**		
How happy are you?	-	44.8%
Your physical health?	-	38.3%
Your work situation?	-	32.5%
Your family’s financial situation?		38.3%
**Other**		
Are you concerned about having cerebral palsy?	65.6%	72.1%

^a^ ≥7 (1 = no pain at all, 9 = a lot of pain)

**Table 5:** Mean and SD of standardised t-values using KIDSCREEN-27 and comparison between adolescents with CP and controls. Higher scores indicate better HRQoL (range 0–100, mean = 50 SD = 10).

		Self-report				Proxy-report		
*KIDSCREEN-27 dimensions*	Adolescents with CP *n* = 64 Mean (SD)	Controls *n* = 173 Mean (SD)	Mean difference (95% CI)	*p*-value	Adolescents with CP *n* = 154 Mean (SD)	Controls *n* = 173 Mean (SD)	Mean difference (95% CI)	*p*-value
Total score	34.2 (5.7)	46.0 (7.8)	11.9 (10.1 to 13.7)	**<0.0001**	29.1 (7.4)	43.9 (7.5)	14.8 (13.1 to 16.4)	**<0.0001**
Physical wellbeing	33.8 (13.5)	49.6 (15.0)	15.8 (11.6 to 20.0)	**<0.0001**	28.2 (9.9)	44.9 (9.9)	16.7 (14.5 to 18.8)	**<0.0001**
Psychological wellbeing	42.5 (7.8)	50.3 (9.9)	7.8 (5.1 to 10.5)	**<0.0001**	34.2 (10.9)	50.5 (11.5)	16.3 (13.9 to 18.8)	**<0.0001**
Autonomy & parents	39.4 (7.6)	44.4 (8.9)	5.0 (2.5 to 7.4)	**<0.0001**	33.3 (10.3)	41.3 (9.6)	8.0 (5.8 to 10.1)	**<0.0001**
Peers & social support	33.5 (8.1)	39.6 (8.6)	6.1 (3.7 to 8.6)	**<0.0001**	25.9 (12.2)	38.2 (10.5)	12.3 (9.8 to 14.8)	**<0.0001**
School environment	48.7 (10.6)^a^	53.0 (9.0)^b^	4.3 (0.7 to 7.8)	**0.012**	44.9 (10.6)^c^	50.4 (9.6)^d^	5.5 (2.1 to 9.0)	**0.003**

^a^ *n* = 33;

^b^ *n* = 152;

^c^ n = 39;

^d^ n = 154

#### Access to rehabilitation

Access to rehabilitation services was limited; only 27.9% of adolescents with CP (according to proxy report) had ever received any rehabilitation service. Service types included therapy, assistive device and advice. Reasons for having never received service were being unaware (50.0%), no money (15.6%), difficulty accessing services (3.9%), transport problems (1.3%) and not requiring services (1.3%). 54.7% of adolescents with CP and 66.2% of proxies were ‘unhappy’ or ‘very unhappy’ about service access. Rehabilitation access was not recorded for Controls due to absence of impairments.

#### Health perception

The majority of adolescents with CP self-reported health to be ‘poor’ or ‘fair’ (56.3%), remainder were ‘good’, ‘very good’ or ‘excellent’. Proxy-report estimated higher proportions of poor or fair health (81.8%). Overall, health perception of adolescents with CP was significantly poorer than for controls (*p*<0.05).

#### CPQoL-Teens

CPQoL-Teens was administered to adolescents with CP only, see Table 3. Adolescents with CP self-reported CPQoL-Teens scores ranging from 57.7 (25.5) to 74.3 (14.3). Proxies reported scores ranging from 38.5 (27.4) to 63.5 (18.3). Dimensions with poorest scores for both self- and proxy-report were ‘feelings about functioning’ followed by ‘general wellbeing and participation’. Best performing dimension for self-report was ‘social wellbeing’ and for proxy-report was ‘school wellbeing’ followed by ‘social wellbeing’. Due to missing scores ‘school wellbeing’ was weighted for non-school attendance; mean scores then decreased to 36.9 (8.3) for self-report and to 16.1 (4.6) for proxy-reports.

### Individual item analysis

Individual item analysis revealed numerous areas of concern for adolescents with CP, see Table 4. Notably, 95.1% of adolescents with CP self-reported a pain rating of ≥7 (1 = no pain, 9 = a lot of pain) as did 82.3% of proxies.

High proportions (≥ 50%) of adolescents with CP self-reported feeling ‘unhappy’ / ‘very unhappy’ (i.e. rated ≤ 3 out of 9; 1 = very unhappy; 9 = very happy) on four items. 65.6% ‘about having CP’, 62.5% about ‘hanging out on their own’, 56.3% about ‘the way they use their legs’ and 51.6% about ‘what may happen to them later in life’. Conversely, high proportions (≥ 50%) of adolescents with CP reported being ‘happy’ or ‘very happy’ (i.e. rated ≥7 out of 9) on 35 items. Of these, 89.1% were ‘happy or very happy’ about ‘the support they get from their family’, 87.6% about ‘succeeding in the things they want to be good at’, 87.5% about ‘the way they get along with the person who looks after them’, 84.4% about ‘how they are accepted by their family’, 82.9% about ‘the way they get along with their brothers and sisters’, 82.9% about ‘the way they communicate with people they know well, 82.8% about ‘being able to do things they want to do’, 78.1% about their ‘ability to get around their neighbourhood’, 76.6% about ‘hanging out with friends’, 76.6% about ‘how they sleep’, and 76.5% about ‘going on trips with their family’.

More than 50% of proxies reported their adolescent to be ‘unhappy’ / ‘very unhappy’ on 27 items. Of these 72.1% were ‘unhappy’ / ‘very unhappy’ about ‘having cerebral palsy’, 72.1% about ‘what may happen to them later in life’, 71.4% about ‘their future’ and 67.5% about ‘life as a whole’. More than 50% of proxies reported ‘happy’ or ‘very happy’ on 15 items. Of these, 70.8% were ‘happy’ or ‘very happy’ about ‘how they are accepted by their family’, 64.2% about ‘the way they get along with their brothers and sisters’, 63.0% about ‘the support they get from their family’, 63.0% about ‘the way they communicate with people they know well’ and 60.3% about ‘going on trips with the family’.

#### Kidscreen-27

KIDSCREEN-27 was administered to adolescents with CP and controls, see Table 5. Adolescents with CP self-reported scores ranging from 33.5 (8.1) to 48.7 (10.6). These scores were significantly poorer than controls, mean difference 4.3 (95% CI 0.7 to 7.8 to 15.8 (95% CI 11.6 to 20.0), (*p*<0.05). Proxies reported scores ranging from 25.9 (12.2) to 44.9 (10.6). Adolescent with CP scores were significantly poorer than for controls, mean difference 5.5 (95% CI 2.1 to 9.0) to 16.7 (95% CI 14.5 to 18.8), (*p*<0.05). The poorest performing dimension for all groups (i.e. cases and controls, self- and proxy-report) was ‘peers and social support’. The best performing dimension for all groups, with exception of controls proxy-report was ‘school environment’. However, when ‘school environment’ scores were weighted to account for missing scores caused by non-school attendance the mean score reduced to 10.4 (2.3) for self-report cases, 46.5 (7.9) for self-report control, 11.4 (2.7) for proxy-report cases, and 44.8 (8.6) for proxy-report control; ‘psychological wellbeing’ then became the best performing dimension for all groups.

### Individual item analysis

Individual item analysis revealed that a high proportion (i.e. ≥ 50%) of adolescents with CP self-reported ‘never’ or ‘seldom’ on 12 items, see Table 6. In particular ‘in the past week’ two-thirds or more of adolescents with CP self-reported having ‘never’ or ‘seldom’ been ‘able to run well’, ‘had enough money to do the same things as their friends’, ‘had enough money for their expenses’, that ‘they or their friends helped each other’, or having ‘been able to rely on friends.

**Table 6:** KIDSCREEN-27 individual item analysis: Proportion of respondents reporting ‘never’ or ‘seldom’ / ‘not at all’ or ‘slightly’.

	Self-report		Proxy-report	
Kidscreen-27 item	Adolescents with CP	Controls	Adolescents with CP	Controls
**Physical wellbeing**				
In general how would you say your health is? ^a^	56.3%	32.9%	81.8%	46.8%
Have you felt fit and well?	20.3%	9.8%	31.2%	4.0%
Have you been physically active (e.g. running, climbing, biking)?	56.9%	23.1%	66.9%	19.1%
Have you been able to run well?	70.3%	18.5%	78.6%	12.1%
Have you felt full of energy?	56.3%	17.3%	77.3%	19.7%
**Psychological wellbeing**				
Has your life been enjoyable	29.7%	11.0%	51.3%	8.7%
Have you been in a good mood?	26.6	12.7%	50.0%	9.2%
Have you had fun?	50.0%	26.6%	63.0%	17.3%
Have you felt sad	11%	2.9%	18.8%	4.6%
Have you felt so bad that you didn’t want to do anything?	3.1%	2.3%	14.3%	2.3%
Have you felt lonely?	15.6%	4.6%	14.9%	2.3%
Have you been happy with the way you are?	42.2%	11.0%	53.9%	5.2%
**Autonomy & parents**				
Have you had enough time for yourself?	35.9%	26.6%	30.5%	20.2%
Have you been able to do the things that you want to do in your free time?	43.8%	28.9%	61.0%	24.3%
Have your parent(s) had enough time for you?	34.4%	26.6%	31.2%	25.4%
Have your parent(s) treated you fairly?	10.9%	2.9%	19.5%	5.2%
Have you been able talk to your parent(s) when you wanted to?	18.8%	15.6%	33.8%	15.0%
Have you had enough money to do the same things as your friends?	84.4%	61.3%	90.3%	78.0%
Have you had enough money for your expenses?	85.9%	65.3%	91.6%	79.8%
**Peers & social support**				
Have you spent time with your friends?	54.7%	30.1%	66.9%	27.7%
Have you had fun with your friends?	57.8%	31.2%	72.1%	28.9%
Have you and your friends helped each other?	70.3%	36.4%	85.7%	58.4%
Have you been able to rely on your friends?	71.9%	37.6%	81.2%	57.8%
**School environment**				
Have you been happy at school?	33.3%	3.3%	41.0%	3.9%
Have you got on well at school?	45.5%	3.3%	10.3%	1.3%
Have you been able to pay attention?	57.6%	7.2%	20.5%	5.2%
Have you got along well with your teachers?	15.2%	5.3%	23.1%	7.1%

^a^ poor/ fair;

Conversely, a high proportion of respondents indicated favourable outcomes on 5 items. 78.1% of adolescents with CP self-reported ‘very often’ or ‘always’ being ‘treated fairly by their parents’, 57.8% as having been able to ‘talk to their parents when they wanted to’, 66.7% as being ‘very’ or ‘extremely happy’ at school, 54.6% as getting on well at school and 54.5% as getting along well with teachers.

Fifteen items were reported as ‘never’ or ‘seldom’ by more than 50% of proxy-reporters. Notably, in the last week 91.6% ‘never’ or ‘seldom’ had ‘enough money for their expenses’, 90.3% ‘enough money to do the same things as their friends’, 85.7% had ‘they and their friends helped each other’, 81.2% been ‘able to rely on their friends’, 78.6% been ‘able to run well’, 77.3% ‘felt full of energy’, 72.1% had ‘fun with their friends’, 66.9% been ‘physically active’, and 66.9% ‘spent time with their friends’. Only one item was reported as ‘very often’ or ‘always’ by more than 50% of proxies; 57.8% of proxies reported ‘very often’ or ‘always’ had their child ‘felt that his/ her parents treated him/ her fairly’.

### Strengths and Difficulties Questionnaire

Mean SDQ of adolescents with CP was significantly poorer than for controls in both self- and proxy-report groups, self-report ‘total difficulties’ score mean difference 5.8 (95% CI 4.4 to 7.1), proxy-report 7.8 (95% CI 6.7 to 8.9), *p*>0.05, see Table 7. Poorest performing dimensions were ‘hyperactivity’ and ‘emotional problems’.

**Table 7:** Mean (SD) and mental health status using Strengths and Difficulties Questionnaire and comparison between adolescents with CP and controls. Higher mean scores indicate better wellbeing. Total difficulties (0–40), Emotional, Conduct, Hyperactivity, Peer, Prosocial (0–10).

SDQ dimensions	Self-report					Proxy-report				
	Adolescents with CP *n* = 64	Control *n* = 173	Odds ratio (95% CI)	Mean difference (95% CI)	*p*-value	Adolescents with CP *n* = 154	Control *n* = 173	Odds ratio (95% CI)	Mean difference (95% CI)	*p*-value
Total difficulties										
*Mean(SD)*	25.2 (5.0)	30.9 (4.6)		5.8 (4.4 to 7.1)	**<0.001**	21.6 (5.2)	29.41 (5.1)		7.8 (6.7 to 8.9)	**<0.001**
*Unlikely (n[%])*	37 (57.8%)	155 (89.6%)	0.2 (0.1 to 0.3)			26 (16.9%)	131 (75.7%)	0.1 (0.0 to 0.1)		
*Possible (n[%])*	15 (23.4%)	13 (7.5%)	3.8 (1.7 to 8.5)			30 (19.5%)	20 (11.6%)	1.9 (1.0 to 3.4)		
*Probable (n[%])*	12(18.8%)	5 (2.9%)	7.8 (2.6 to 23.0)			98 (63.6%)	22 (12.7%)	12.0 (6.9 to 20.9)		
Emotional problems										
*Mean(SD)*	5.5 (1.9)	7.4 (2.0)		1.9 (1.3 to 2.5)	**<0.001**	4.4 (2.2)	6.59 (2.1)		2.2 (1.7 to 2.6)	**<0.001**
*Unlikely (n[%])*	41 (64.1%)	157 (90.8%)	0.2 (0.1 to 0.4)			37 (24.0%)	94 (54.3%)	0.3 (0.2 to 0.4)		
*Possible (n[%])*	16 (25.0%)	8 (4.6%)	6.9 (2.8 to 17.0)			10 (6.5%)	28 (16.2%)	0.4 (0.2 to 0.8)		
*Probable (n[%])*	7 (10.9%)	8 (4.6%)	2.5 (0.9 to 7.3)			107 (69.5%)	51 (29.5%)	5.4 (3.4 to 8.7)		
Conduct problems										
*Mean(SD)*	7.6 (1.7)	8.3 (1.4)		0.7 (0.3 to 1.1)	**0.002**	7.1 (1.7)	8.06 (1.5)		1.0 (0.6 to 1.3)	**<0.001**
*Unlikely (n[%])*	49 (76.6%)	153 (88.4%)	0.4 (0.2 to 0.9)			81 (52.6%)	118 (68.2%)	0.5 (0.3 to 0.8)		
*Possible (n[%])*	6 (9.4%)	14 (8.1%)	1.2 (0.4 to 3.2)			25 (16.2%)	32 (18.5%)	0.8 (0.5 to 1.5)		
*Probable (n[%])*	9 (14.1%)	6 (3.5%)	4.6 (1.6 to 13.4)			48 (31.2%)	23 (13.3%)	3.0 (1.7 to 5.1)		
Hyperactivity										
*Mean(SD)*	5.5 (1.9)	7.0 (1.8)		1.3 (0.7 to 1.8)	**<0.001**	4.1 (2.2)	6.88 (1.9)		2.8 (2.3 to 3.2)	**<0.001**
*Unlikely (n[%])*	47 (73.4%)	161 (93.1%)	0.2 (0.1 to 0.5)			64 (41.6%)	153 (88.4%)	0.1 (0.1 to 0.2)		
*Possible (n[%])*	13 (20.3%)	5 (2.9%)	8.6 (2.9 to 25.2)			25 (16.2%)	11 (6.4%)	2.9 (1.4 to 6.0)		
*Probable (n[%])*	4 (6.3%)	7 (4.1%)	1.6 (0.4 to 5.6)			65 (42.2%)	9 (5.2%)	13.3 (6.3 to 28.0)		
Peer problems										
*Mean(SD)*	6.2 (1.7)	8.1 (1.4)		1.9 (1.5 to 2.3)	**<0.001**	6.0 (1.9)	7.87 (1.5)		1.9 (1.5 to 2.3)	**<0.001**
*Unlikely (n[%])*	26 (40.6%)	148 (85.6%)	0.1 (0.1 to 0.2)			36 (23.4%)	115 (66.5%)	0.2 (0.1 to 0.3)		
*Possible (n[%])*	31 (48.4%)	22 (12.7%)	6.4 (3.3 to 12.5)			22 (14.3%)	27 (15.6%)	0.9 (0.5 to 1.7)		
*Probable (n[%])*	7 (10.9%)	3 (1.7%)	7.0 (1.7 to 27.8)			96 (62.3%)	31 (17.9%)	7.6 (4.6 to 12.6)		
Prosocial										
*Mean(SD)*	6.9 (2.4)	8.7 (1.4)		1.8 (1.3 to 2.3)	**<0.001**	5.2 (2.9)	8.47 (1.8)		3.3 (2.8 to 3.8)	**<0.001**
*Unlikely (n[%])*	13 (20.3%)	0 (0.0%)	-			68 (44.2%)	7 (4.1%)	18.8 (8.3 to 42.6)		
*Possible (n[%])*	6 (9.4%)	3 (1.7%)	5.9 (1.4 to 24.2)			10 (6.5%)	7 (4.1%)	1.6 (0.6 to 4.4)		
*Probable (n[%])*	45 (70.3%)	170 (98.3%)	0.4 (0.0 to 1.4)			76 (49.4%)	159 (91.9%)	0.1 (0.0 to 0.2)		

Adolescents with CP showed high proportions of ‘probable’ mental health problems including emotional, conduct, hyperactivity and peer problems. Higher proportions of problems were reported in the proxy group than the self-report group. Odds of adolescents with CP reporting ‘probable’ range total difficulties was 7.8 (95% CI 2.6 to 23.0) and 12.0 (95% CI 6.9 to 20.9), self- and proxy-report, respectively. Encouragingly, a high proportion of adolescents with CP reported ‘probable’ range prosocial behaviour indicating positive outcomes on this domain, although they were more likely than controls to report ‘unlikely’. 0% of controls self-reported unlikely and hence OR could not be calculated. Odds of adolescents with CP reporting ‘unlikely’ in proxy report was 18.8 (95% CI 8.3 to 42.6).

## Discussion

To the best of our knowledge, our study is one of the first studies from LMICs which looked at the HRQoL and mental health of adolescents with CP from a population-based sample and compared that with age and sex matched controls. Moreover, we have used multi-respondent assessment and utilised both general population (KDSCREEN) and CP-specific tools (CPQoL) for measuring HRQoL. We found that the HRQoL of adolescents with CP was significantly poorer than for age and sex matched peers without disability. Analysis of HRQoL dimensions revealed that ‘feelings about functioning’ for CPQoL-Teens and ‘peers and social support’, followed closely by ‘physical wellbeing’ for KIDSCREEN-27 were the poorest dimensions overall for adolescents with CP. Highest scores, indicating better HRQoL, were observed in ‘social wellbeing’ for CPQoL-Teens and ‘psychological wellbeing’ for KIDSCREEN-27. In regards to mental health, adolescents with CP reported significantly poorer outcomes than controls on all dimensions. The dimensions ‘hyperactivity’ and ‘emotional’ problems were poorest overall and ‘conduct problems’ was highest overall for adolescents with CP.

Initially, high scores were observed in ‘school’ dimensions for CPQoL-Teens and KIDSCREEN-27, although results were biased by missing scores that corresponded approximately to non-school attendance. Bangladesh has endorsed ‘education for all’ policies although access to education for adolescents with disability is a challenge [24]. Only 25.3% of adolescents with CP attended school compared with 96.3% of controls; adolescents with CP who attended school tended to report lower GMFCS level and absence of cognitive and speech impairments. ‘School wellbeing’ scores were notably good for those who attended school.

When dimension comparison was conducted between CPQoL-Teens and KIDSCREEN-27 the outcomes of dimensions related to social wellbeing appeared contradictory. Specifically, ‘social wellbeing’ scored highest on CPQoL-Teens whereas KIDSCREEN-27 ‘peers and social support’ reported lowest scores. Qualitative analysis of the instrument items revealed differ theoretical approaches between the instruments. CPQoL-Teens focused on how adolescents feel, for example “how do you feel about the way you get along with people generally” whereas KIDSCREEN-27 focused on activities and outcomes “thinking about the last week have you spent time with your friends”. We were unable to locate studies comparing CPQoL-Teens and KIDSCREEN-27 outcomes, although discordance between thematically related dimensions of other HRQoL instruments have been reported for similar reasons [25].

We could not find any studies on adolescents with CP from LMICs using CPQoL-Teens or KIDSCREEN-27. However, our findings of disproportionately poorer HRQoL amongst adolescents with CP were in keeping with research from other LMICs using instruments such as the Child Health Questionnaire [26] and Paediatric Quality of Life Inventory [27]. These studies reported physical wellbeing to be the poorest dimension of HRQoL. To the best of our knowledge SDQ has never been used among children and adolescents with CP in LMICs. However, SDQ has previously been used in Bangladesh among general child population, and our results indicated higher rates of mental health problems among adolescents with CP although self-reported SDQ of controls was comparable [22].

Numerous studies on HRQoL and mental health have been conducted in HICs and our findings are converse to some but not all [28]. For example, a large European study found similar wellbeing amongst adolescents with CP and their peers without disability using KIDSCREEN-52 on all dimensions except ‘social support and peers’ [8]. Whereas an American study using the Paediatric Quality of Life inventory reported children and adolescents with CP to have poorer outcomes on all dimensions [29]. Our findings of mental health also differed to other studies. A systematic review reported pooled prevalence of SDQ total difficulties score at 35% [11]; our findings were lower for self-reported outcomes and higher for proxy-report. Overall our findings were poorer than normative data for KIDSCREEN-27 [30] and Australian Data for SDQ [31]. Normative data was not yet available for CPQoL-Teens.

A limitation of this study is that all instruments had originally been developed in predominately high-income countries in Europe and Australia and may be culturally bound to their country of origin, and that age application of CPQoL-Teens and SDQ differed from our sample (i.e. 13 to 18y and 4 to 17y respectively, vs our inclusion of ≥10 to ≤18y). We included adolescents from 10 years of age in our sample as this is considered a normative classification of adolescence in Bangladesh [15]. Moreover, we conducted rigorous forward and backward cross-cultural translation and psychometric validation of CPQoL-Teens and Kidscreen-27 to confirm their suitability to assess HRQoL in 10 to 18 year olds in Bangladesh. The instruments performed well psychometrically [18, 21] and both instruments reflected internationally defined multidimensional theoretical constructs of HRQoL although we were unable to account for how HRQoL was conceptualised amongst adolescents with CP in rural Bangladesh. Future research may address this gap. SDQ had previously been tested and confirmed as psychometrically suitable to assess adolescent mental health in Bangladesh [22].

The UNCRPD has recommended that people with disability, including adolescents, have their voices heard on matters that affect their lives [13]. Whist involvement of adolescents with CP in the design of this research was not feasible; our approach ensured that wherever possible adolescents self-reported their HRQoL. An inbuilt challenge to this approach, and limitation in our research, is that adolescents with communication or cognitive impairment may be excluded from self-reporting. This was the case in our sample, only 64 cases (i.e. 41.6% of the cohort) provided self-reported HRQoL and were significantly less likely to have speech or cognitive impairment. These differences in adolescent characteristics can partially explain higher HRQoL in self-reported compared to proxy-reported outcomes. Moreover, adolescents and their caregivers are likely to have different reasoning processes when answering questions [32]. Research has consistently shown limited agreement between adolescent and proxy-reported perspectives with more agreement on observable physical dimensions and lesser agreement on psychological and social dimensions [32].

Adolescents with CP in Bangladesh face numerous difficulties that are likely to impact their HRQoL and mental health. For example, lack of access to assistive devices such as wheelchairs can compound the impact of impairment and contribute to social isolation. Moreover, financially or geographically inaccessible medical services may lead to unmanaged comorbidities including pain and epilepsy; physical and attitudinal barriers may result in adolescents being excluded from or unable to get to school (i.e. poor roads, inaccessible transport, unwilling or unskilled teachers) and participate in community life; small or overcrowded homes and limited sanitation can mean that hygiene is a particular challenge for those requiring support with personal care, especially for adolescent girls after menarche, and negative beliefs about disability can threaten individuals physical safety [12]. Adolescents will no doubt be able to identify personal strengths and positive experiences in their lives, although sadly, our findings indicated high amounts of pain, social isolation, and fear about the future.

This research has provided important insight into the HRQoL of adolescents with CP in rural Bangladesh although further work is necessary to comprehensively understand HRQoL in this LMIC context. For example, exploration of how HRQoL is conceptualised in Bangladesh and if CPQoL-Teens and KIDSCREEN-27 adequately capture these concepts; increased sample size, conduct of HRQoL and mental health research focused on adolescents with CP in other districts of Bangladesh; and improved strategies for data collection directly from adolescents with speech impairment and/or cognitive disability will strengthen future research in this area.

## Conclusion

This study represents our first understanding of the HRQoL and mental health of adolescents with CP from rural Bangladesh and can provide guidance on priority areas for resource allocation and intervention. Our findings identified areas of concern for adolescents with CP in regards to HRQoL and mental health and that outcomes were significantly poorer than for age and sex matched peers without disability. High proportions of adolescents with CP reported having a lot of pain, and concerns about friendships, physical activity and energy, what may happen later in life, and feelings about having CP. Intervention to reduce disparity in HRQoL and mental health is required and resources should be targeted to improve physical, psychological, and social wellbeing to enhance long-term HRQoL and mental health of adolescents with CP in LMICs like Bangladesh. Specific interventions to alleviate modifiable aspects of HRQoL including pain, social isolation, and physical in-activity are recommended.

## Supporting information

S1 Table Study tools and procedures. (DOC)

## Abbreviations

CP: Cerebral palsy
CPQoL-Teens: Cerebral palsy quality of life teenager questionnaire
GMFCS: Gross motor function classification system
HIC: High income country
HRQoL: Health-related quality of life
LMIC: Low and middle-income country
SD: Standard deviation
SDQ: Strengths and difficulties questionnaire
