# Supplementary material for: Health-related quality of life and mental health of adolescents with cerebral palsy in rural Bangladesh
Source: PLoS One. 2019 Jun 11;14(6):e0217675. doi: 10.1371/journal.pone.0217675 (PMC6561392; doi:10.1371/journal.pone.0217675)
Supplement: S1 Table — (DOC) [file pone.0217675.s001.doc]

## Suppl. study material 1: Study tools and procedures

|  | Case (n=154) | | Control (n=173) | |
| --- | --- | --- | --- | --- |
|  | Adolescent with CP who could self-report | Primary caregiver [Proxy report] | Adolescent without disability self-report | Primary caregiver [Proxy report] |
| Cerebral Palsy Quality of Life Questionnaire for Teens (CPQoL-Teens) | ✔ [n=64] | ✔[n=154] | - | - |
| KIDSCREEN-27 Health Questionnaire | ✔ [n=64] | ✔[n=154] | ✔ [n=173] | ✔[n=173] |
| Strengths and Difficulties Questionnaire (SDQ) | ✔ [n=64] | ✔[n=154] | ✔ [n=173] | ✔[n=173] |
